# Integrated metabolomics and gut microbiota analysis to explore the potential mechanism of levo-tetrahydropalmatine against ketamine addiction

**DOI:** 10.3389/fphar.2025.1698866

**Published:** 2025-11-19

**Authors:** Yan Du, Qing Ma, Xingcui Gao, Hongliang Su, Keming Yun, Li Du

**Affiliations:** 1 School of Pharmacy, Shanxi Medical University, Taiyuan, Shanxi, China; 2 China Institute of Radiation Protection, Taiyuan, Shanxi, China; 3 School of Forensic Medicine, Shanxi Medical University, Taiyuan, Shanxi, China; 4 Department of Pharmacy, Shanxi Province Cancer Hospital, Taiyuan, Shanxi, China

**Keywords:** L-tetrahydropalmatine, ketamine addiction, metabolomics, gut microbiota, correlation analysis

## Abstract

**Introduction:**

Metabolic and gut microbiota (GM) disturbance play a significant role in the complex pathogenesis of substance dependence. Although levo-tetrahydropalmatine (l-THP) demonstrates therapeutic potential in drug addiction, the underlying mechanism remains elusive.

**Methods:**

The efficacy of l-THP was assessed using a conditioned place preference (CPP) paradigm. In this research, we combined metabolomics with gut microbiota analysis to investigate the potential mechanisms underlying l-THP’s intervention in ketamine (KET) addiction.

**Results:**

L-THP effectively relieved hippocampus (Hip) pathological changes and attenuated KET-induced rewarding effects. Metabolomic profiling of serum and urine samples revealed a total of 194 distinct metabolites, including 3-methoxytyramine, 3-hydroxyphenylacetic acid, and phosphorylcholine, involving glycerophospholipid metabolism, tyrosine metabolism, phenylalanine metabolism, dopaminergic synapse, teichoic acid biosynthesis, staurosporine biosynthesis, and lysine biosynthesis metabolism pathways. L-THP also enriched *Firmicutes, Lactobacillus*, *Dubosiella*, unclassified_*Clostridia*_UCG_014, and *Ligilactobacillus* while decreasing the levels of *Bacteroidota*, *Campylobacterota*, *Patescibacteria*, unclassified_*Bacilli*, UCG_005, *Prevotella*, and *Romboutsia.* Moreover, Spearman’s correlation analysis showed that discriminative metabolites were closely correlated with special bacteria.

**Conclusion:**

The findings demonstrated that l-THP might serve as a promising intervention strategy for KET addiction by regulating serum and urine metabolism and gut microbiota.

## 1 Introduction

Ketamine (KET) was initially utilized in surgical procedures because of its anesthetic, sedative, and pain-relieving effects. KET became a popular street drug since 1970 (World Health Organization, 2012). KET is regarded as a widely abused drug among teenagers (Weir, 2000), which is manifested in family problems, lost productivity, and crime. The prolonged use of KET leads to sustained modifications in behavior and brain structures related to learning and memory (Duman, 2002). The learned associations between signals and the positive reinforcement effects of abused drugs create memories that are difficult to erase (Hyman, et al., 2006). Studies demonstrated that the mesolimbic dopamine system plays a crucial role in influencing behavioral and neuronal alterations induced by drugs (Volkow, et al., 2009). So far, there is no effective medicine to control the high relapse of KET. Therefore, there is an urgent need for exploring the underlying mechanisms of KET dependence and to find effective treatment medicine.

Levo-tetrahydropalmatine (l-THP), derived from the plants *Corydalis ambigua* and *Stephania tetrandra*, exhibits a range of pharmacological effects, including sedation, neuroleptic action, and analgesia (Zhang et al., 2018). Hence, l-THP has been used as a medication to alleviate pain and induce sedation in China for many years. Pharmacological studies have shown that l-THP acts as an inhibitor of dopamine D1 and D2 receptors. L-THP exhibits a comparatively reduced binding affinity toward dopamine D_3_ receptors (Su et al., 2020). The characteristics of l-THP indicate that it could potentially offer therapeutic benefits for drug addiction (Wang and Mantsch, 2012). L-THP can reduce methamphetamine-induced locomotor sensitization (Zhao et al., 2014), conditioned place preference (CPP) (Su et al., 2013), oxycodone-induced CPP (Liu et al., 2009), and cocaine self-administration (Mantsch et al., 2010). Our previous study indicated that l-THP can attenuate the rewarding behavior of KET (Du et al., 2017). However, the mechanism by which l-THP regulates metabolism and gut microbiota (GM) of KET addiction remains unclear.

Metabolomics investigates the endogenous metabolites present in organisms, including their composition, levels, and alterations. Metabolomics can speculate prospective metabolic pathways and screen biomarkers, offering valuable insights into the therapeutic mechanisms of medications. At present, studies have shown that drug addiction causes changes in metabolites, including KET, methylamphetamine, and morphine addiction (Zaitsu et al., 2014), and traditional Chinese medicine (TCM) can intervene drug addiction by modifying the metabolic disorders (Zhu et al., 2017). Metabolomic analysis of blood and urine components in animals can offer valuable insights into TCM treatment of drug addiction.

At present, research on the mechanisms of addiction has focused on the central nervous system (CNS). Nevertheless, research has shown the existence of the brain–gut–microbiota (BGM) axis in humans, indicating a two-way interaction between the brain and gut microbiota, which implies that the gut microbiota can affect the functioning of the host’s brain (Cryan and Dinan, 2012; Osadchiy et al., 2019). Studies indicated that gut microbiota play a crucial role in drug addiction and are more disrupted in rats with drug abused than in healthy rats (García-Cabrerizo et al., 2021). Studies have shown that TCM can treat drug addiction by modifying the composition of the gut microbiota (Zhuang et al., 2024). Therefore, combining metabolomic analysis with 16S rRNA sequencing can help elucidate the intervention mechanism of TCM in treating KET addiction.

This research investigated the intervention effects of l-THP and its fundamental mechanisms in a rat model of CPP. Hippocampal neuron damage caused by addiction was determined. The integration of nontargeted metabolomic profiling combined with 16S ribosomal RNA gene sequencing was utilized to investigate the differential metabolites, metabolic processes, and gut microbiota, associated with efficacy. Moreover, the findings can serve as a foundation for l-THP intervention and the treatment of KET addiction.

## 2 Materials and methods

### 2.1 Materials and chemicals

L-THP (99.00%) was purchased from Sigma-Aldrich Inc. (United States). KET hydrochloride was manufactured by Heng-Rui pharmaceutical factory, Jiangxi, China. The volume of intraperitoneal (i.p.) injection was 10.0 mL/kg.

### 2.2 Animal and treatment

Male Sprague–Dawley (SD) rats (6–8 weeks) were purchased from SPF Biotechnology Co., Ltd., animal license number: SCXK (Beijing) 2019-0007, under standard conditions (22 °C ± 2 °C, 55% ± 5% humidity, and 12-h light/dark cycle). They had unrestricted access to food and water and underwent a 1-week acclimation period. Approval for all animal-related experimental procedures was obtained from the Institutional Animal Care and Use Committee of Shanxi Medical University.

The rats were divided into three groups: (1) control group administrated with saline (n = 10, control group), (2) ketamine (10 mg/kg) treatment group (n = 10, KET group), and (3) L-THP (20 mg/kg) combined with ketamine (10 mg/kg) treatment group (n = 10, K + T group). Ketamine was administered via intraperitoneal injection, whereas l-THP was given by oral gavage. L-THP was administrated 30 min before KET administration. Based on our previous study demonstrating that 20 mg/kg l-THP reversed KET-induced CPP (Du et al., 2017), this single dose was selected.

### 2.3 CPP instrument and experiment

CPP chambers (30 × 30 × 40 cm), comprising black and white compartments separated by a removable door, were utilized to evaluate addiction behavior (Du et al., 2020).

**FIGURE 1:**
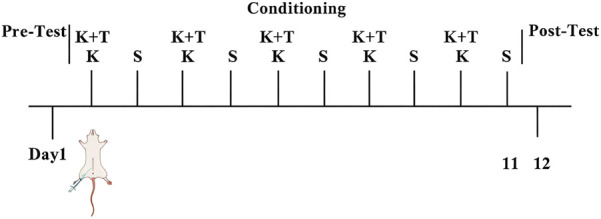
CPP behavioral schedule. K, KET group; S, control group; K + T, K + T group.

Day 1: rats freely explored both compartments for 15 min, and the initial preference (DigBehv system) defined the drug-paired compartment (least time spent). During the conditioning phase (days 2, 4, 6, 8, and 10), the KET group received KET (10 mg/kg i. p.), whereas the control group received saline. The K + T group received l-THP 30 min prior to KET. Immediately after injection, rats were confined to the drug-paired (white) compartment for 40 min. On days 3, 5, 7, 9, and 11, all groups received saline and were confined to the opposite (black) compartment for 40 min. CPP expression was tested on day 12 (15 min free access, compartment times recorded via DigBehv) (Figure 1).

### 2.4 Sample collection

After the post-test of CPP on day 12, rats were anesthetized, and blood was collected from the abdominal aorta under anesthesia. The samples were kept at room temperature for 1 h, then centrifuged (3000 rpm, 10 min, 4 °C), followed by a second centrifugation (12,000 rpm, 10 min, and 4 °C). Urine samples were collected via metabolic cages and centrifuged (1500 g, 10 min, and 4 °C). Fresh colonic contents were collected into sterile tubes. The supernatant serum and colonic contents were stored at −80 °C.

### 2.5 Assessment of KET addiction

Rats’ body weights were measured every 2 days. Following CPP, the rats were anesthetized and perfused with 4% paraformaldehyde and 0.9% saline. Brain samples were post-fixed overnight, embedded in paraffin, sectioned at 4 μm, and stained with hematoxylin–eosin (H&E) for histological evaluation.

### 2.6 Metabolomic analysis

#### 2.6.1 Sample preparation

Samples (100 μL blood or urine) were mixed with 500 μL extraction solution containing internal standard (methanol: acetonitrile = 1:1, 20 mg/L internal standard) and vortexed for 30 s. Blood samples were then sonicated for 10 min (ice-water bath); urine samples were directly analyzed using LC/MS.

#### 2.6.2 UPLC-Q-TOF-MS conditions

The analysis used an ultra-high-performance liquid chromatography system (Waters Acquity I-Class PLUS) coupled to a Waters Xevo G2-XS QTOF high-resolution mass spectrometer. Separation was achieved using a Waters Acquity UPLC HSS T3 column (2.1 mm × 100 mm, 1.8 µm particle size). For both positive and negative ionization modes, the mobile phases consisted of (A) 0.1% formic acid in water and (B) 0.1% formic acid in acetonitrile. Sample injection volumes were 1 µL for serum and 2 µL for urine.

Mass spectrometry was performed using a Waters Xevo G2-XS QTOF high-resolution instrument. Collision energy was set to 2 V (low) and ramped from 10 to 40 V (high), with a scan time of 0.2 s per spectrum. ESI source parameters were as follows: capillary voltage: +2000 V (positive)/−1500 V (negative); cone voltage: 30 V; source temperature: 150 °C; desolvation temperature: 500 °C; cone gas flow: 50 L/h; desolvation gas flow: 800 L/h.

#### 2.6.3 Data preprocessing and statistical analysis

Raw data (MassLynx V4.2) were processed using Progenesis QI software for peak extraction, alignment, and metabolite identification against the online METLIN database and Biomark’s self-built library. Theoretical fragment identification and mass deviation were constrained to <0.1 Da. Peak areas were normalized to total areas prior to analysis.

Sample repeatability and QC performance were assessed via principal component analysis (PCA) and Spearman correlation. Identified metabolites were annotated using the Kyoto Encyclopedia of Genes and Genomes (KEGG) and Pathways Strategy (lipid maps) databases for classification and pathway analysis. For differential analysis, fold change (FC) and significance (*p*-value, *t*-test) were calculated per compound based on group information. Orthogonal projections to latent structures–discriminant analysis (OPLS-DA) were performed using R, with variable importance in projection (VIP) values derived from multiple cross-validation. Differential metabolites were screened using combined thresholds: *p* < 0.05 and VIP >1. Significant KEGG pathway enrichment was determined using the hypergeometric distribution test.

### 2.7 Gut microbiota analysis

An analysis was conducted on changes in GM using the high-throughput 16S rRNA sequencing technology. The quality and quantity of the extracted DNA from the colonic contents were examined using electrophoresis on a 1.8% agarose gel, and DNA concentration and purity were determined using a NanoDrop 2000 UV-VIS spectrophotometer (Thermo Scientific, Wilmington, United States). By using the template DNA, alongside primers containing unique barcodes, gene amplification was achieved through the polymerase chain reaction (PCR). After quantification, the NovaSeq 6000 platform was used for sequencing the amplicons (Beijing Biomarker Technologies Co., Ltd., Beijing, China). QIIME2 2020.6 software (version 1.9.1) was used to perform quality filtering on the sequence reads.

### 2.8 Correlation analysis between serum metabolites and gut microbiota

In our study, serum metabolite–gut microbiota relationships were analyzed using the Pearson correlation coefficient, with correlation values of *r* > 0.7 considered statistically significant.

### 2.9 Correlation analysis between urine metabolites and gut microbiota

In our study, urine metabolite–gut microbiota relationships were analyzed using the Pearson correlation coefficient, with correlation values of *r* > 0.7 considered statistically significant.

### 2.10 Statistical analysis

CPP scores = (post-test − pre-test time in drug-paired chamber) (Li et al., 2008). Statistical analyses were performed using SPSS 16.0 (SPSS, Inc., Chicago, Il, United States). Multigroup comparisons were conducted by one-way ANOVA with the Bonferroni *post hoc* test. Data were presented as mean ± SEM; a *p*-value <0.05 was considered significant.

## 3 Results

### 3.1 The effect of l-THP on KET-induced CPP

**FIGURE 2:**
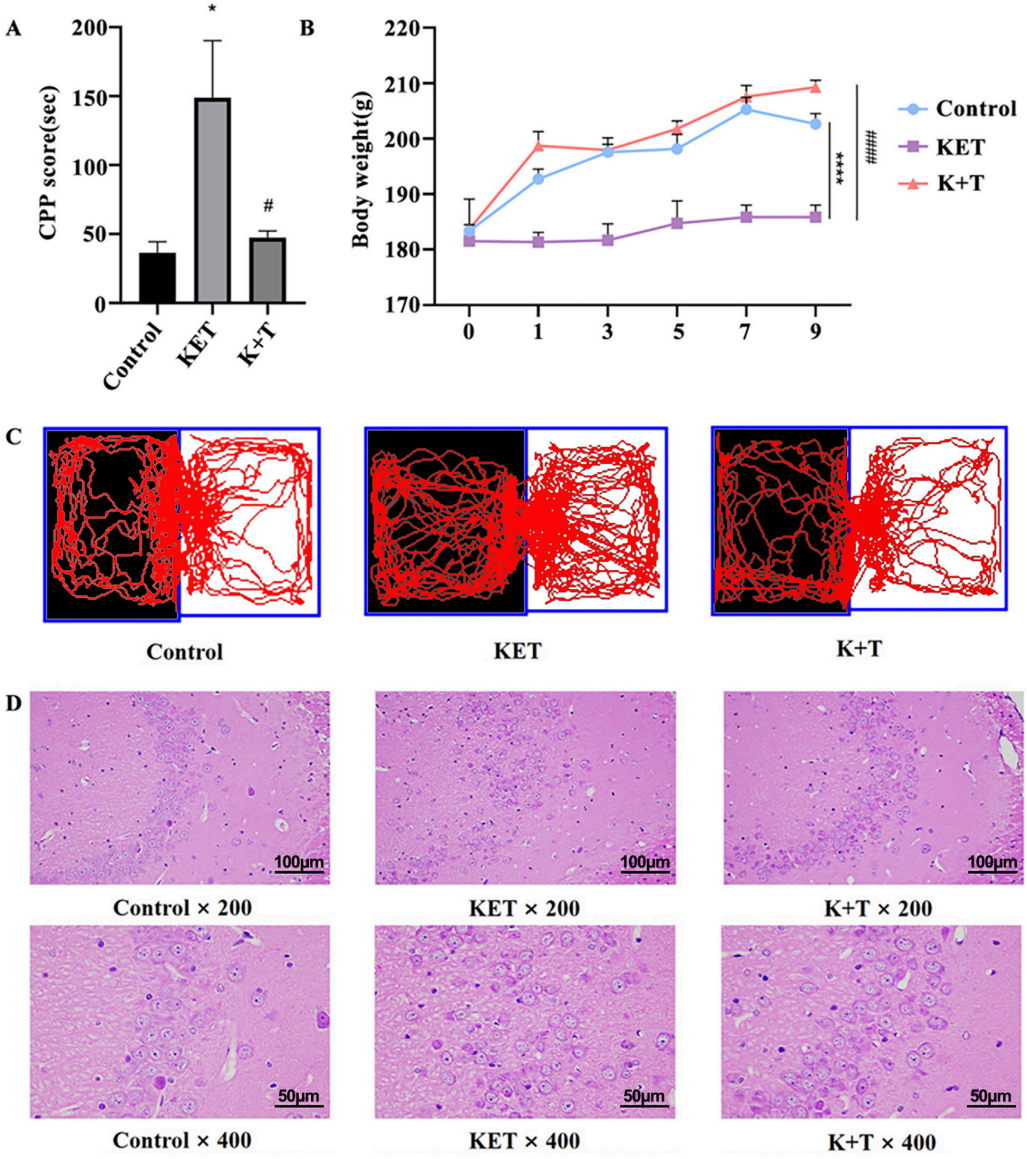
Assessment of KET addiction in rats. **(A)** CPP scores. **(B)** Body weight changes in rat. **(C)** Visual representations depicting the trajectories followed by rats within the CPP compartments. **(D)** L-THP alleviates Hip pathological injury from KET-induced CPP rats. HE staining of Hip tissues in Control, KET, and K + T groups (×200 and ×400, scale bar; 100 μm and 50 µm). The data are presented as means ± SEM (n = 10 per group). *p* < 0.05 was considered statistically significant. KET group vs. control group, ^*^ *p* < 0.05; KET group vs. K + T group, ^#^ *p* < 0.05.

As shown in Figures 2B,C, KET induced a significant preference for the drug-associated environment, with a mean conditioning score of 148.8 ± 16.9 s. Pretreatment with l-THP significantly inhibited this effect, reducing the mean conditioning score to 47.3 ± 12.1 s [F (2, 15) = 6.38, ^#^ *p* < 0.05]. L-THP treatment significantly decreased the time spent in the white box compared to the KET group. Body weight changes were used as a physiological indicator of KET addiction. We measured the body weights of the rats every 2 days (Figure 2A). No significant weight differences were observed on day 0. However, by day 9, rats in the KET group exhibited significant weight loss compared to those in the control group, whereas those in the K + T group showed a more gradual weight gain. H&E staining of Hip CA3 tissues revealed that KET exposure led to loosen pyramidal cell arrangements, disordered architecture, irregular shapes, nuclear condensation, and cytoplasmic shrinkage. In contrast, the K + T group showed neatly arranged pyramidal cells with intact structure, suggesting that l-THP effectively mitigates Hip injury induced by KET (Figure 2D).

### 3.2 L-THP regulated metabolic profiles in KET-induced CPP rats

#### 3.2.1 Metabolic profiling analysis

**FIGURE 3:**
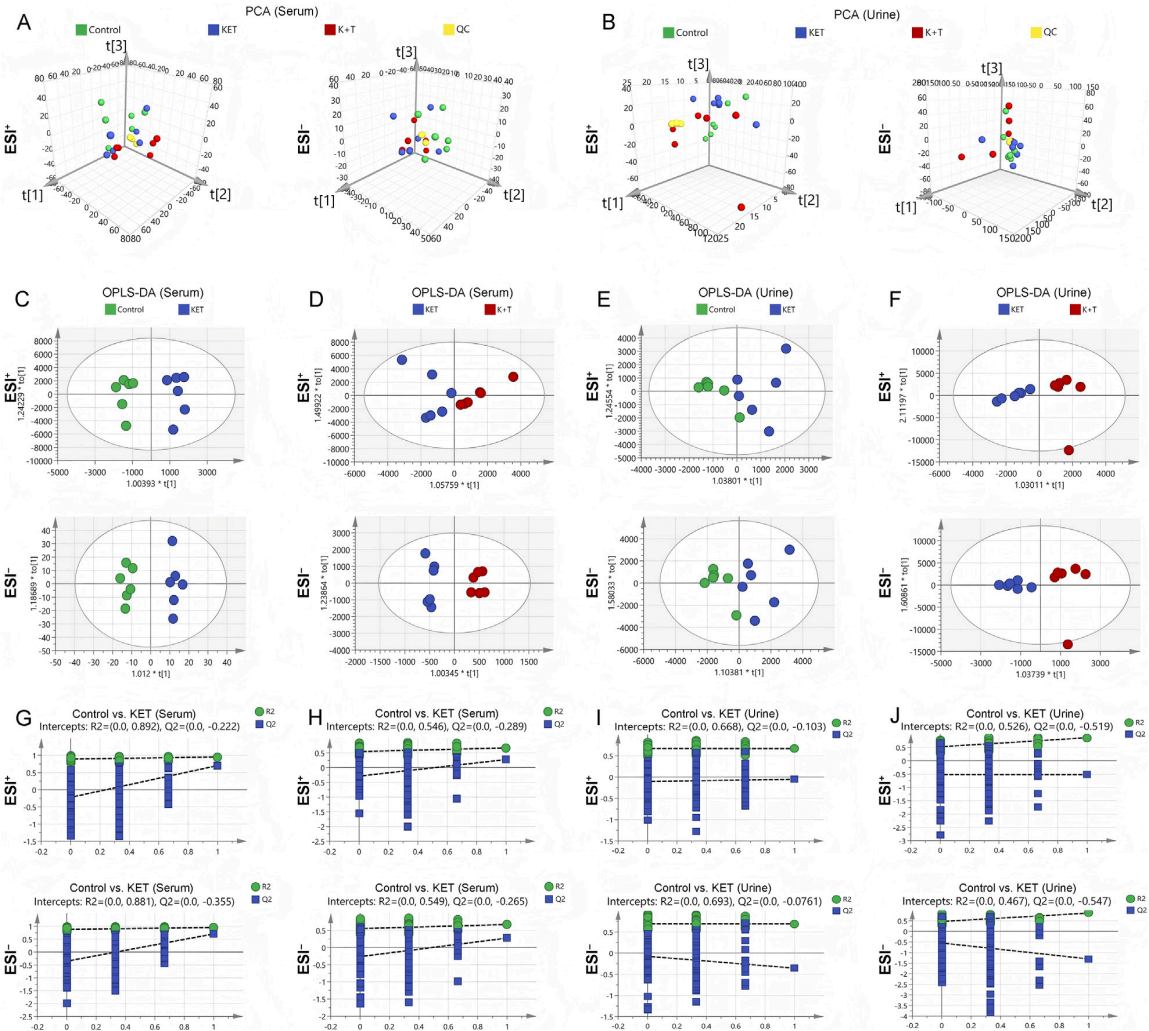
Multivariate analysis of serum/urine metabolome: PCA: serum **(A)** and urine **(B)**; OPLS-DA score plots: serum **(C,D)** and urine **(E,F)**; permutation tests: serum **(G,H)** and urine **(I,J)**.

To investigate the potential therapeutic mechanisms of l-THP, an untargeted metabolic profiling analysis was conducted on serum and urine samples from the control, the KET, and the K + T groups. As shown in Figures 3A,B, in the PCA score plots of both serum and urine samples, the QC samples clustered closely, demonstrating good stability and reproducibility of the analytical platform. As the control, KET, and K + T groups did not show clear separation in the PCA results, an OPLS-DA model was subsequently constructed to further investigate the metabolic differences among the groups. The resulting OPLS-DA plot revealed a clear distinction between the control and KET groups in both the serum and urine metabolomes. Additionally, a noticeable separation was observed between the K + T group and the KET group (Figures 3C–F). Permutation tests demonstrated that the OPLS-DA model was both reliable and free from overfitting, distinguishing among the control, KET, and K + T groups (Figures 3G–J). The findings indicated that l-THP effectively modulated metabolic disturbances in rats exhibiting KET-induced CPP.

#### 3.2.2 Screening and identification of differential metabolites

**FIGURE 4:**
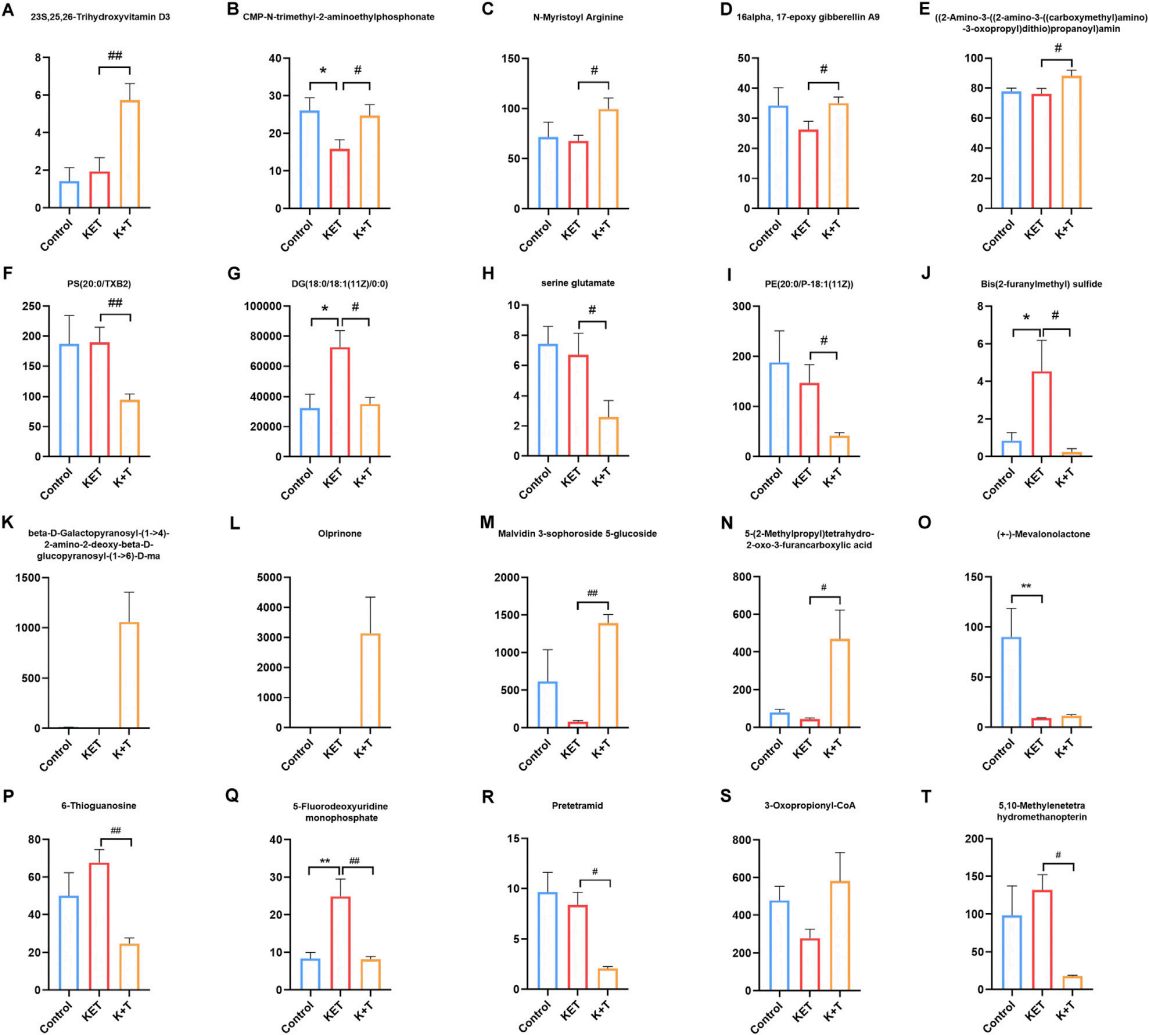
**(A-T)** Levels of 20 differential metabolites between K + T and KET groups. Compared with Control group, *p < 0.05, **p < 0.01. Compared with KET group, #*p* < 0.05, ##*p* < 0.01.

The initial screening of differential metabolites was conducted using a threshold of VIP >1 and *p* < 0.05, as determined by Student’s t-test. Through database search and literature review, 66 metabolites showed differences in serum samples, and 128 metabolites showed differences in urine samples (Supplementary Material S1). The top 20 differential metabolites between the KET and K + T groups in serum and urine are shown in Table 1 and Figure 4.

**TABLE 1:** Identification and trends of differential metabolites in KET-abused rats after l-THP treatment.

No	Metabolite	HMDB number	Molecular formula	MW	VIP	FC	p-value	K + T vs. KET	RT	Source
1	23S,25,26-Trihydroxyvitamin D3	HMDB0060134	C_27_H_44_O_4_	432.6	2.639	2.966	0.008	↑^**^	9.49	serum
2	CMP-N-trimethyl-2-aminoethylphosphonate	HMDB0060072	C_14_H_27_N_4_O_10_P_2_^+^	473.3	2.358	1.556	0.044	↑^*^	0.81	serum
3	N-Myristoyl Arginine	HMDB0242046	C_20_H_40_N_4_O_3_	384.6	2.187	1.468	0.036	↑^*^	9.39	serum
4	16alpha, 17-epoxy gibberellin A9	HMDB0304030	C_19_H_23_O_5_^−^	331.4	2.099	1.336	0.031	↑^*^	2.59	serum
5	((2-Amino-3-((2-amino-3-((carboxymethyl)amino)-3-oxopropyl)dithio)propanoyl)amino)acetic acid	HMDB0242125	C_10_H_18_N_4_O_6_S_2_	354.4	2.094	1.155	0.046	↑^*^	0.03	serum
6	PS (20:0/TXB2)	HMDB0282083	C_46_H_84_NO_14_P	906.1	2.817	0.498	0.011	↓^*^	10.26	serum
7	DG (18:0/18:1 (11Z)/0:0)	HMDB0007159	C_39_H_74_O_5_	623.0	2.843	0.483	0.018	↓^*^	9.40	serum
8	Serine glutamate	HMDB0028828	C_8_H_14_N_2_O_7_	250.2	2.001	0.385	0.048	↓^*^	7.81	serum
9	PE (20:0/P-18:1 (11Z))	HMDB0009248	C_43_H_84_NO_7_P	758.1	2.458	0.282	0.035	↓^*^	10.19	serum
10	Bis (2-furanylmethyl) sulfide	HMDB0041503	C_10_H_10_O_2_S	194.3	2.421	0.053	0.049	↓^*^	3.78	serum
11	beta-D-Galactopyranosyl-(1->4)-2-amino-2-deoxy-beta-D-glucopyranosyl-(1->6)-D-mannose	HMDB0041224	C_18_H_33_NO_15_	504.2	2.158	2330487162.46	0.049660886	↑^*^	2.20	urine
12	Olprinone	HMDB0255959	C_14_H_10_N_4_O	518.2	2.175	39254.76	0.049	↑^*^	2.77	urine
13	Malvidin 3-sophoroside 5-glucoside	HMDB0242046	C_35_H_45_O_22_^+^	816.2	2.214	11.78	0.041	↑^*^	2.89	urine
14	5-(2-Methylpropyl) tetrahydro-2-oxo-3-furancarboxylic acid	HMDB0030988	C_9_H_14_O_4_	231.1	2.247	10.67	0.039	↑^*^	4.70	urine
15	(+-)-Mevalonolactone	--	C_6_H^[3]^2H^[1]^_8_O_3_	265.1	2.584	10.27	0.014	↑^*^	6.03	urine
16	6-Thioguanosine	HMDB0247107	C_10_H_13_N_5_O_4_S	616.2	2.355	0.23	0.021	↓^*^	3.10	urine
17	5-Fluorodeoxyuridine monophosphate	HMDB0060394	C_9_H_12_FN_2_O_8_P	371.0	2.059	0.21	0.048	↓^*^	2.45	urine
18	Pretetramid	--	C_19_H_13_NO_6_	396.1	2.357	0.17	0.027	↓^*^	2.51	urine
19	3-Oxopimeloyl-CoA	HMDB0012158	C_28_H_44_N_7_O_20_P_3_S	965.2	2.047	0.15	0.050	↓^*^	3.11	urine
20	5,10-Methylenetetrahydromethanopterin	HMDB0060401	C_31_H_45_N_6_O_16_P	823.2	2.469	0.11	0.021	↓^*^	2.77	urine

#### 3.2.3 Metabolic pathway analysis

**FIGURE 5:**
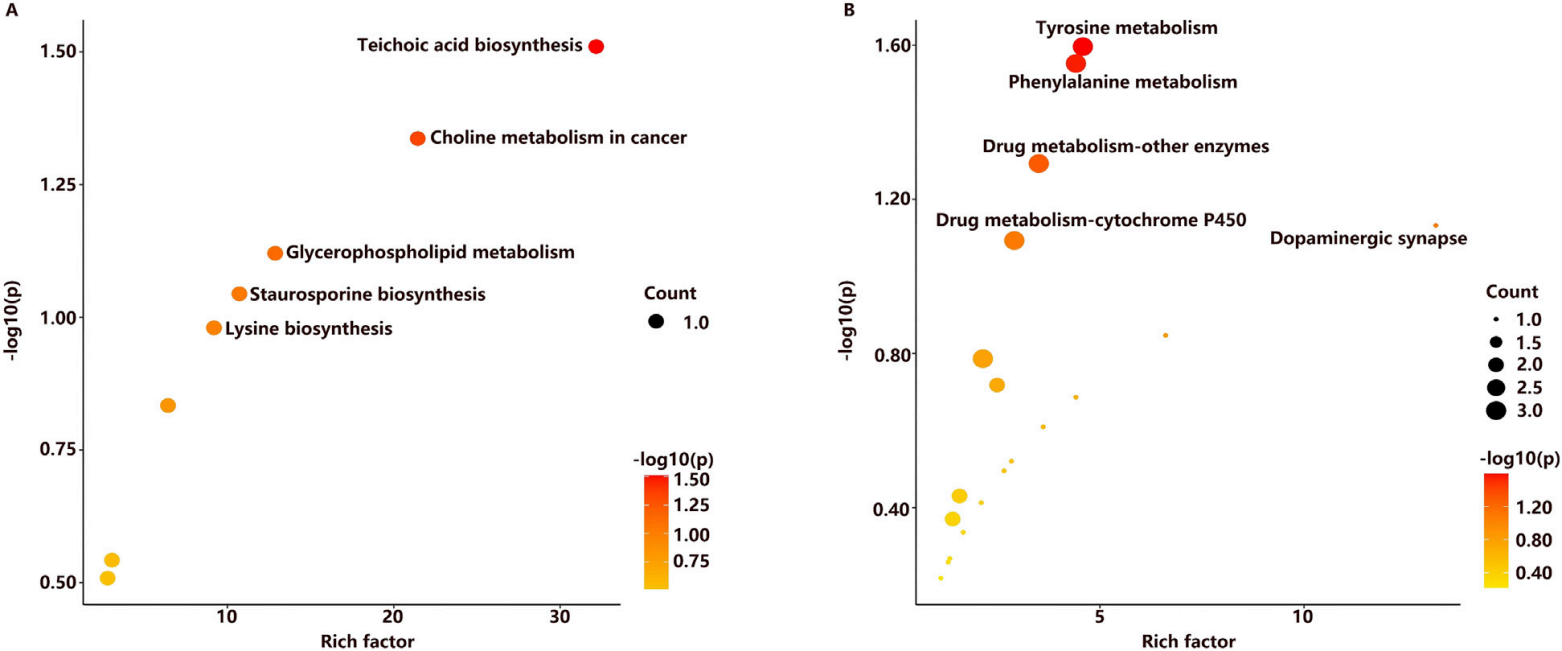
Metabolic pathways related to l-THP treatment in serum **(A)** and urine **(B)**.

**FIGURE 6:**
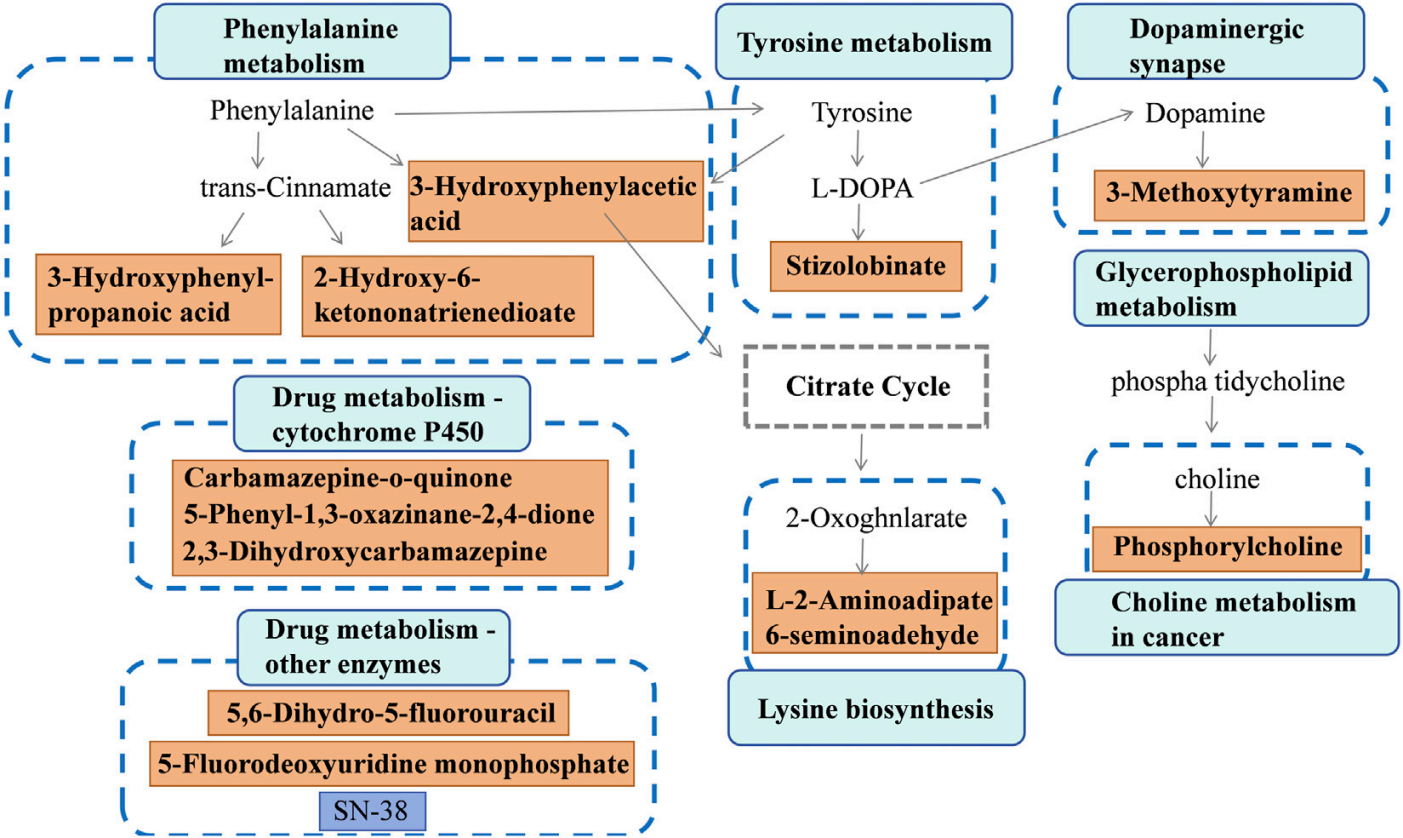
Metabolite network during l-THP modulation: green↑ (upregulated) and red↓ (downregulated).

Discriminative metabolites in serum were mainly involved in glycerophospholipid metabolism, teichoic acid biosynthesis, choline metabolism in cancer, staurosporine biosynthesis, and lysine biosynthesis metabolism (Figure 5A). As for urine samples, the differential metabolites were mainly involved in tyrosine metabolism, phenylalanine metabolism, dopaminergic synapse, drug metabolism by other enzymes, and cytochrome P450 pathways (Figure 5B). A comprehensive summary of the differentially altered metabolites and their related metabolic pathways was established to offer a broader insight into the impact of l-THP on the metabolome (Figure 6).

### 3.3 Gut microbiota analysis

**FIGURE 7:**
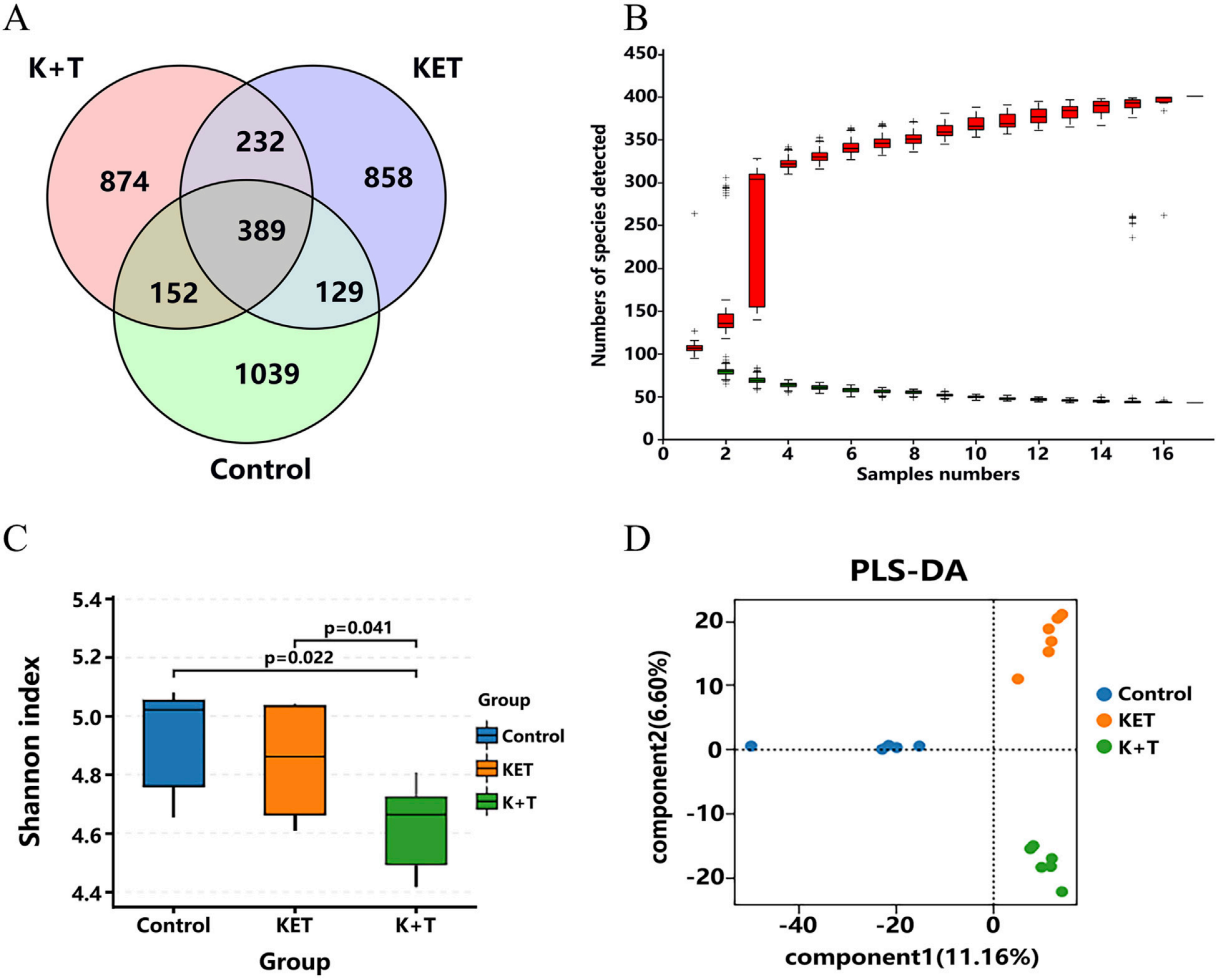
L-THP intervene KET-induced CPP by modulation of GM. **(A)** Venn analysis on intestine microbiota of control, KET, and K + T groups; **(B)** species accumulation curve; **(C)** Shannon diversity indices; **(D)** PLS-DA analysis of intestinal flora in different groups: control group (blue), KET group (orange), and K + T groups (green).

At present, the gut–brain axis is emerging as an important role of shaping behavior and cognition (Cryan, et al., 2019). To explore whether the addiction of KET was associated with gut microbiota, 16S rRNA gene sequences of the rat colonic contents were analyzed after treatment, and the difference was evaluated. In terms of the OTU classification, a comprehensive count of 3,673 genera was identified among the groups. In total, 389 genera were found to overlap across these groups, constituting approximately 10.59% (Figure 7A). In addition, it can be observed from Figure 7B that the species accumulation curves of all 18 samples reached a saturation point, suggesting that the sequencing quantity was sufficient to encompass most species for subsequent analysis. The α-diversity analysis (Figure 7C) indicated a significant decrease in microbiota diversity in the K + T group (*p* < 0.05). PLS-DA analysis revealed a significant dissimilarity in the microbiota composition among the control, KET, and K + T groups, resulting in the formation of three separate clusters (Figure 7D).

**FIGURE 8:**
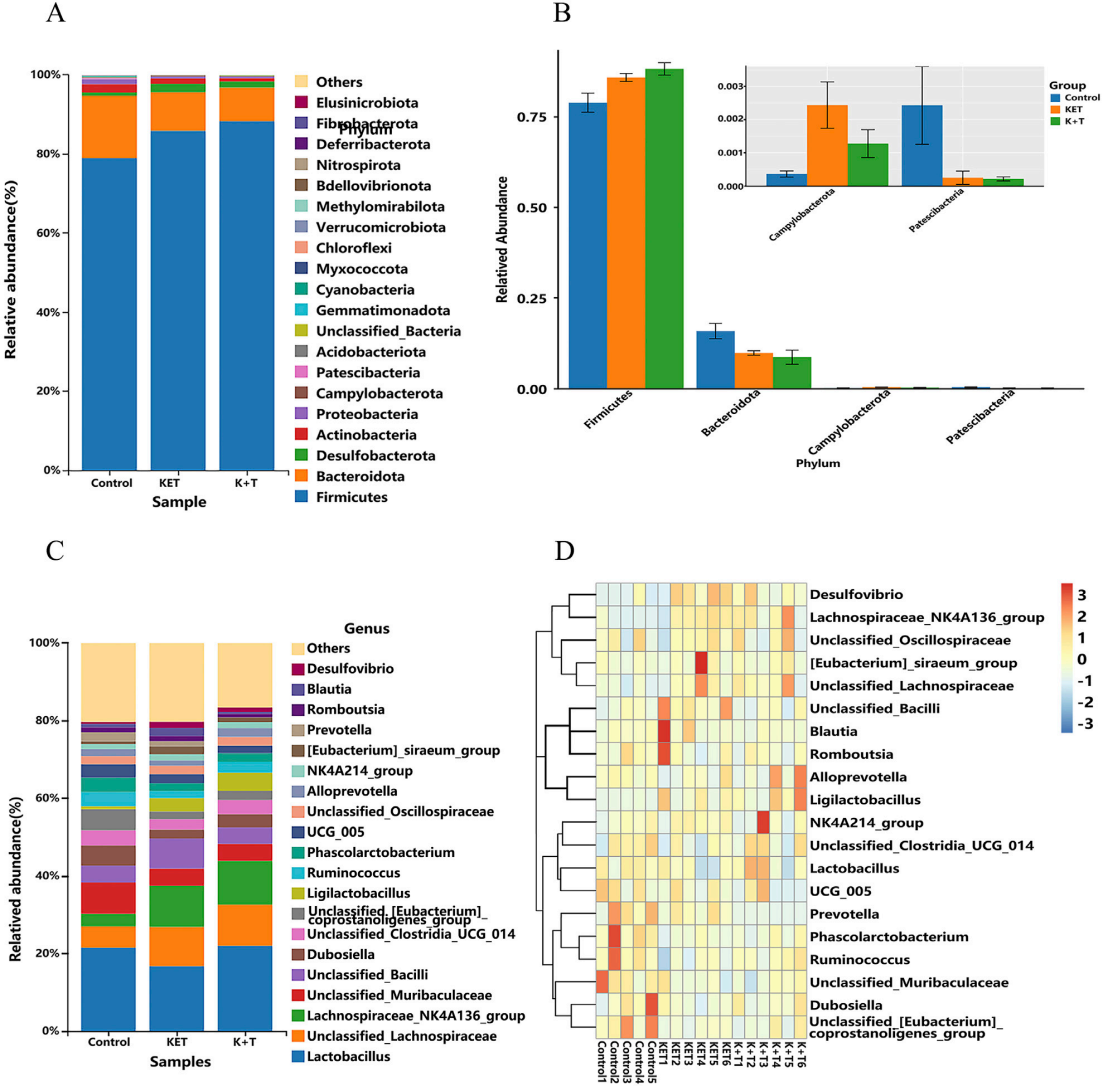
L-THP effects on gut microbiota in KET-abused rats: **(A)** phylum-level abundance distribution. **(B)** Relative abundance differences (Firmicutes, Bacteroidota, Campylobacterota, and Patescibacteria) among groups (mean ± SEM, n = 6). **(C)** Genus-level abundance distribution. **(D)** Heatmap of top 20 genera.

**FIGURE 9:**
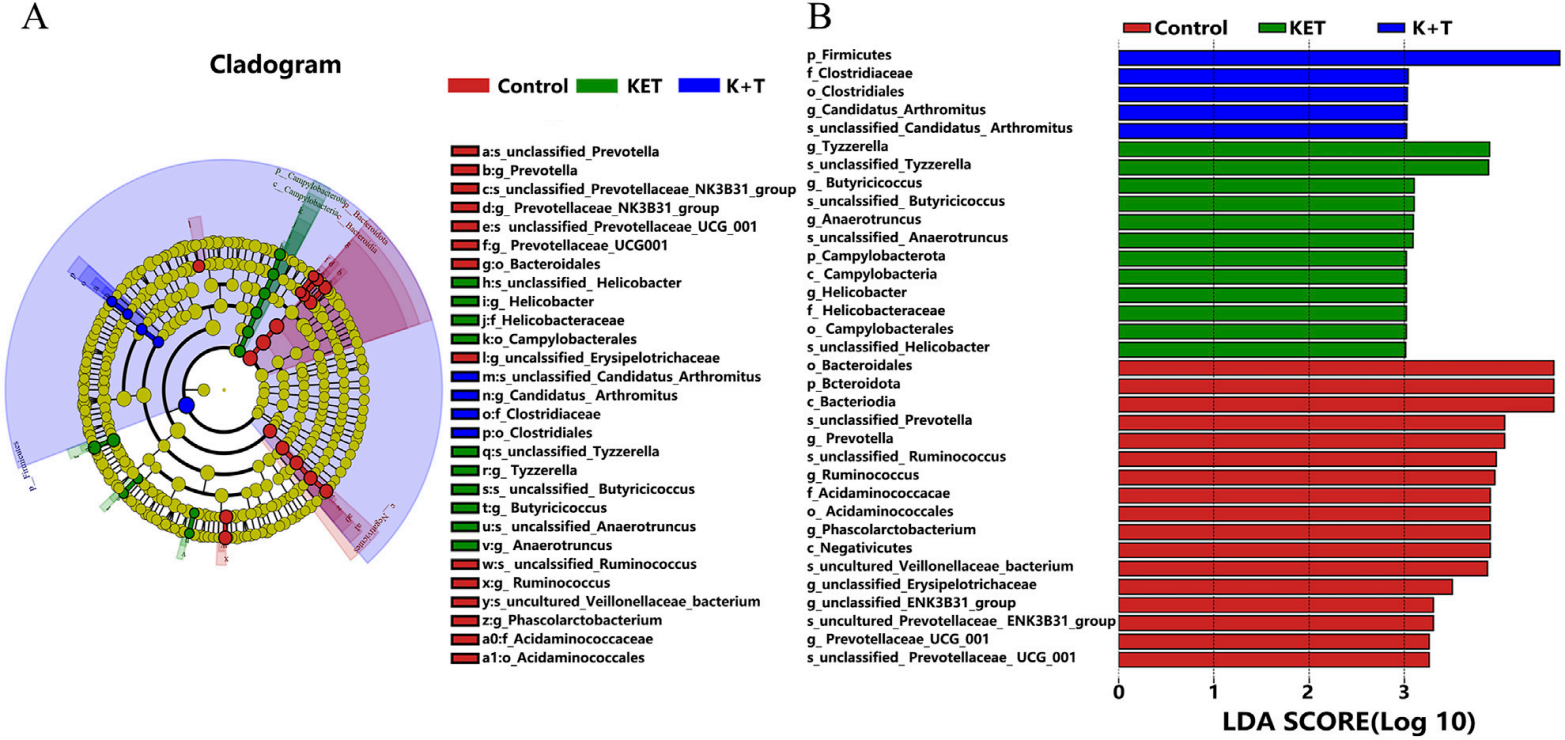
LEfSe analysis of species with significant differences: **(A)** cladogram. **(B)** LDA coupled with effect size measurements identifies the most differentially abundant taxon at the phylum–species level among the control, KET, and K + T groups. Taxa enriched in the control have a positive score (red), KET-enriched taxa are indicated with a positive LDA score (green), and K + T-enriched taxa are indicated with a positive LDA score (blue). Only taxa meeting an LDA significance threshold of >3 are shown.

The histograms of the community (Figures 8A–C) showed that compared to those in the KET group, the changes in community composition and diversity in the K + T group were mainly as follows: at the phylum level, *Firmicutes* in the K + T group increased, and *Bacteroidota*, *Campylobacterota*, and *Patescibacteria* decreased, whereas at the genus level, *Lactobacillus*, *Dubosiella*, unclassified*_Clostridia*_UCG_014, and *Ligilactobacillus* increased, and unclassified*_Bacilli*, *UCG_005*, *Prevotella*, and *Romboutsia* decreased. Meanwhile, a heatmap was used to visualize the 20 most prevalent genera at the genus level (Figure 8D). From the phylum level to the genus level, significant differences were identified using linear discriminant analysis effect size (LEfSe) analysis among colonic contents of the control, KET, and K + T groups (Figure 9A). The predominant genera found in the gut microbiota of the KET group, *p_Campylobacterota*, was found to be the dominant phylum, whereas *g_Tyzzerella*, *g_Butyricicoccus*, *g_Anaerotruncus*, and *g_Helicobacter* were significantly enriched at the genus level. *p_Firmicutes* at the phylum level increased after the administration of l-THP (Figure 9B).

### 3.4 Correlation analysis between serum metabolites and gut microbiota

**FIGURE 10:**
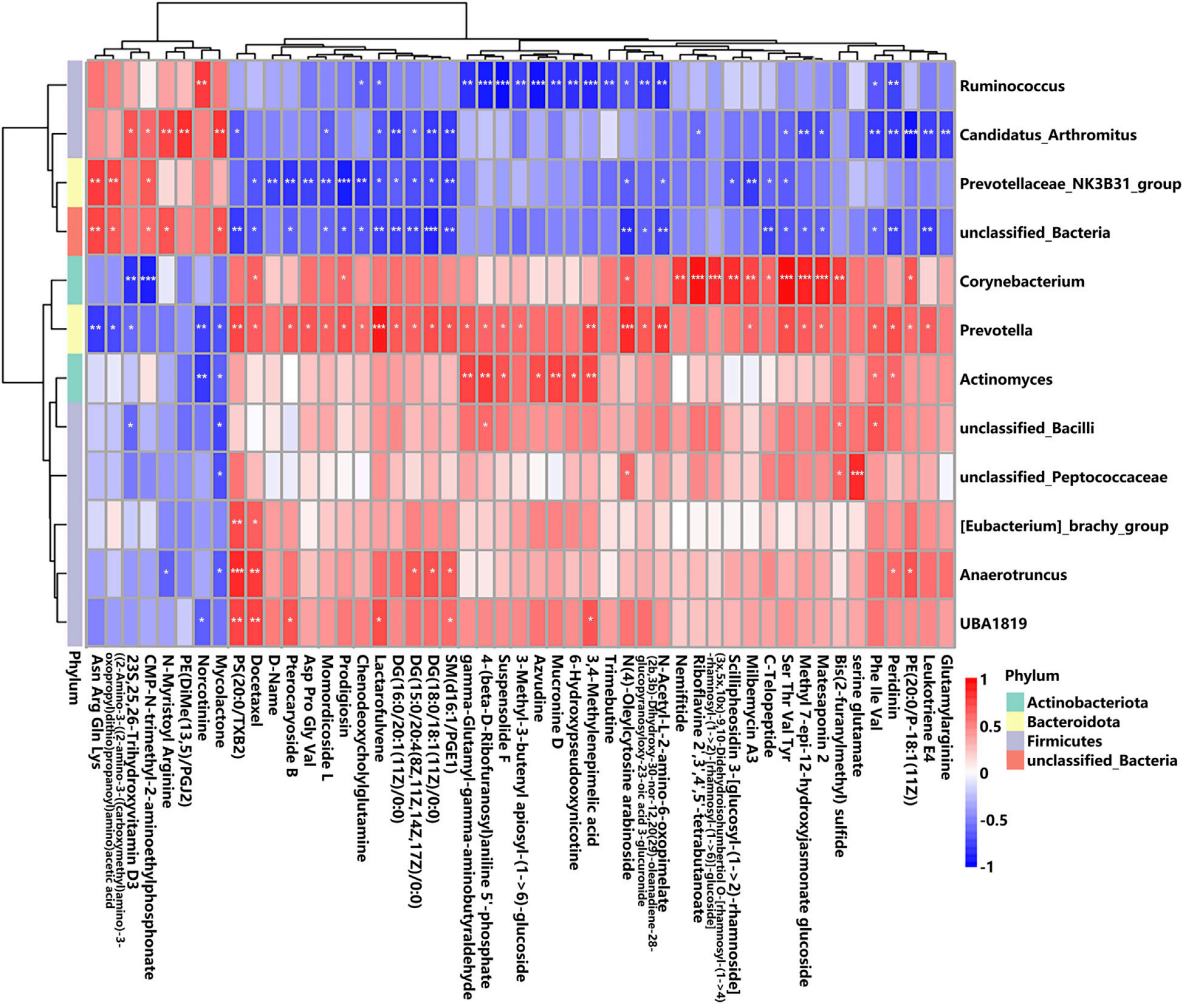
Correlation between altered GM and different serum metabolites. The *r*-value is displayed in different colors, with the red grid indicating a positive correlation and the blue grid showing a negative correlation. The larger the absolute value of *r*, the darker the grid color. ^*^ *p* < 0.05, ^**^ *p* < 0.01, and ^***^ *p* < 0.001.

To reveal the potential connections between metabolites and gut microbiota, Spearman’s rank correlation analysis was calculated. As shown in Figure 10, the associations between the 49 altered metabolites and the 12 altered gut genera were illustrated. The genera enriched in the K + T group (*Ruminococcus*, *Candidatus*_*Arthromitus*, Prevotellaceae_NK3B31_group, and unclassified_*Bacteria*) and those in the KET group (*Corynebacterium*, *Prevotella*, *Actinomyces*, unclassified_*Bacilli*, unclassified_Peptococcaceae, [Eubacterium]_brachy_group, *Anaerotruncus*, and UBA 1819) behaved in the opposite manner (Supplementary Material S2).

Gut microbiota and altered metabolites were grouped into two clusters depending on their correlations. The first metabolite cluster contained eight metabolites, and the second cluster contained 41 metabolites. In total, there were 172 correlations (*p* < 0.05) between the metabolite types and the gut microbiota, of which Prevotellaceae_NK3B31_group was found to be significantly enriched in the K + T group (Supplementary Material S2). Reports demonstrated that Prevotellaceae _ NK3B31_group has a relationship with drug addiction. Therefore, the associated metabolites were studied, and the results were as follows: (H-Asn-Arg-Gln-Lys-OH, r = 0.60, *p* < 0.01; ((2–amino-3-((2-amino-3-((carboxymethyl)amino)-3-oxopropyl)dithio) propanoyl)amino)acetic acid, r = 0.64, *p* < 0.01; D-name, r = −0.61, *p* < 0.01; pterocaryoside B, r = −0.63, *p* < 0.01; Asp Pro Gly Val, r = −0.64, *p* < 0.01; momordicoside L, r = −0.68, *p* < 0.01; prodigiosin, r = −0.83, *p* < 0.001; chenodeoxycholylglutamine, r = −0.86, *p* < 0.01; SM(d16:1/PGE1), r = −0.54, *p* < 0.01; and milbemycin A3, r = −0.67, *p* < 0.01). These relationships suggested that gut microbiota and metabolites were closely related and influenced each other.

### 3.5 Correlation analysis between urine metabolites and gut microbiota

**FIGURE 11:**
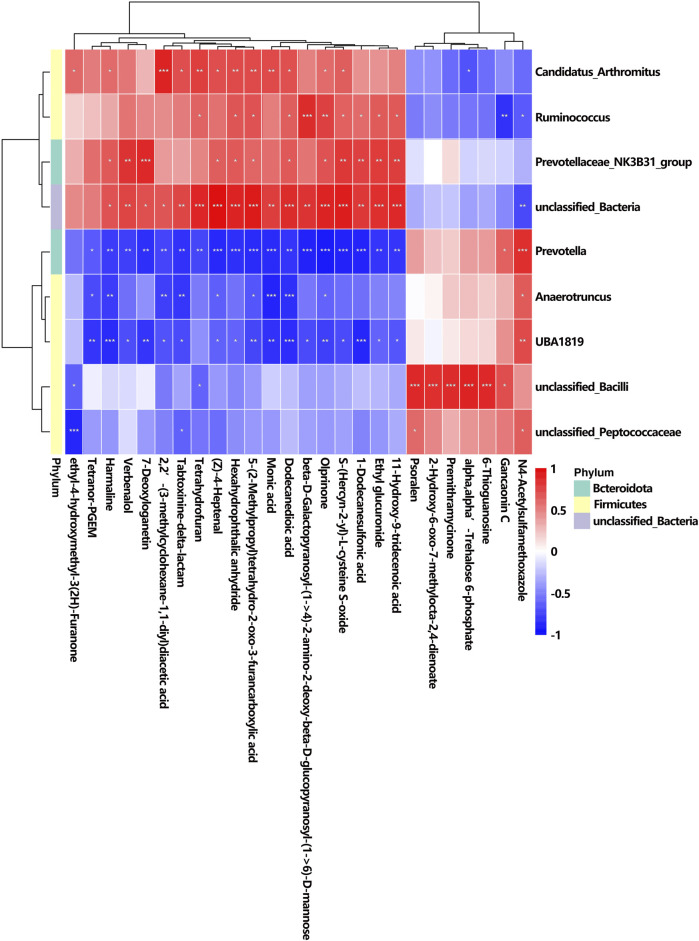
Correlation between altered GM and different urine metabolites. The *r-*value is displayed in different colors, with the red grid indicating a positive correlation and the blue grid showing a negative correlation. The larger the absolute value of r, the darker the grid color. ^*^ *p* < 0.05, ^**^ *p* < 0.01, and ^***^ *p* < 0.001.

The correlation analysis between urine metabolites and gut microbiota depicts the associations identified among nine altered bacterial genera and 26 differentially abundant metabolites, which is consistent with the results of correlation analysis between serum metabolites and gut microbiota. Distinct patterns of enrichment are depicted in Figure 11: genera such as *Candidatus_Arthromitus*, *Ruminococcus,* Prevotellaceae_NK3B31_group, and unclassified_*Bacteria* were more abundant in the K + T group, whereas *Prevotella, Anaerotruncus*, *UBA 181*9, unclassified_*Bacilli*, and unclassified_Peptococcaceae showed enrichment in the KET group, indicating contrasting profiles.

Among the 26 differential metabolites, studies have shown that harmaline is associated with neurological disorders such as addiction and anxiety. Therefore, we specifically highlighted the relationship between harmaline and its associated microbial communities: *Candidatus_Arthromitus*, *r* = 0.58, *p* < 0.05; Prevotellaceae*_NK3B31*_group, *r* = 0.65, *p* < 0.05; unclassified_*Bacteria*, *r* = 0.68, *p* < 0.05; *Prevotella*, *r* = −0.77, *p* < 0.01; *Anaerotruncus*, *r* = −0.77, *p* < 0.01; and *UBA 1819*, *r* = −0.84, *p* < 0.001.

## 4 Discussion

Among the various substances that can disrupt the adolescent brain’s reward system, KET stands out as a particularly harmful, alongside other serious addictions such as alcohol and junk food (Tarantino et al., 2022). Meanwhile, because of its dissociative and hallucinogenic properties, repeated recreational use has been associated with an increasing frequency of hepatotoxicity (Thakkar and Wu, 2025). It is difficult to overcome KET addiction, and there are high rates of relapse after periods of abstinence. L-THP has anti-addictive effects and neuroprotective properties and may play therapeutic and protective roles in KET abuse (Wu, et al., 2007). In this study, we established a rat model with KET-induced CPP. The results indicated that the administration of l-THP significantly attenuated KET-induced CPP.

Metabolic dysfunctions play a significant role in the pathophysiological alterations that occur during the progression of drug addiction. In this study, metabolomic analysis of serum was utilized to identify notable changes in glycerophospholipid metabolites, including phosphorylcholine, among rats in the KET and K + T groups. Phosphorylcholine, a prominent constituent of the glycerophospholipid group, exhibits potential in enhancing the lipid metabolism and addressing cognitive decline. We found that the serum levels of phosphorylcholine exhibited a decrease in rats with KET addiction and l-THP treatment. Glycerophospholipids, being the predominant phospholipid within the human body, serve as a fundamental constituent of cellular membranes (Farooqui, et al., 2000). They have a significant impact on the dynamics of synaptic membranes and work together with synapsins to enhance the exocytosis and endocytosis processes of synaptic vesicles (Mochel, 2018). Significantly, the imbalanced metabolism of glycerophospholipids could potentially contribute to nerve impairment and inflammatory responses (Farooqui, et al., 2007). Therefore, in our study, the disturbance of glycerophospholipid metabolism may lead to KET-induced neuronal damage and inflammation. Research has indicated a correlation between drug addiction and lipid metabolism (Zohairi et al., 2023). The lipidome of the prefrontal cortex and striatum can be significantly altered due to alcohol exposure, resulting in neurotoxicity and neuroplasticity associated with alcohol consumption (Zhang et al., 2022). A recent investigation revealed that the administration of cocaine can induce alterations in certain phospholipids, such as phosphatidylethanolamines (PEs), phosphatidylserines (PSs), and phosphatidylcholines (PCs), within the Hip and cerebellum (Cummings et al., 2015). Hence, the regulation of glycerophospholipid metabolic homeostasis is responsible for the beneficial effects of l-THP in preserving neuronal function, mitigating inflammation and nerve injury, and enhancing synaptic plasticity (Morais et al., 2018). However, the precise mechanism underlying the impact of l-THP on drug dependence via regulating the metabolism of glycerophospholipids remains to be further studied.

In addition, metabolomic analysis of urine was utilized to identify notable changes in tyrosine metabolism and phenylalanine metabolites. Aromatic amino acids consist of phenylalanine, tyrosine, and tryptophan. Phenylalanine can be converted into tyrosine via the action of phenylalanine hydroxylase, and tyrosine can subsequently be metabolized into various neurotransmitters, including dopamine, norepinephrine, epinephrine, and melanin, under the catalysis of tyrosine hydroxylase. Heroin adversely impacts the brain’s reward system by altering the functioning of various neurotransmitters in the central nervous system, including dopamine, gamma-aminobutyric acid (GABA), serotonin (5-HT), and acetylcholine (Leshner, 1997; Tomkins and Sellers, 2001). As precursors to catecholamines, phenylalanine and tyrosine play diverse roles in physiological processes. Disruptions in their metabolism may contribute to neurological deterioration and depressive disorders (Capuron et al., 2011; Hüfner et al., 2019). Hence, the regulation of tyrosine and phenylalanine metabolic homeostasis is responsible for the beneficial effects of l-THP in intervening KET addiction. However, the precise mechanism underlying the impact of l-THP on drug dependence via regulating the metabolism of tyrosine and phenylalanine remains to be further studied.

The treatment with l-THP on KET addiction is related not only to metabolism disorder but also to gut microbiota disorder. The intestinal microbiome significantly contributes to the development and progression of substance dependence. Research demonstrated that the abundance phyla were as follows: *Firmicutes*, *Proteobacteria*, *Actinobacteria*, *Bacteroidete*s, and *Fusobacteria* in methamphetamine groups (Yang et al., 2021). In our study, the top five most abundant phyla were *Firmicutes*, *Bacteroidota*, *Desulfobacterota*, *Actinobacteria*, and *Proteobacteria* in the KET group. The species of phyla we identified are in accordance with previous reports. Studies demonstrated that morphine leads to distinct alterations in the proportions of bacterial populations (Wang et al., 2018). Throughout the duration and dosage of morphine exposure, detrimental bacteria showed an upward trend, whereas beneficial bacteria exhibit a decrease (Sharma et al., 2020). We found that KET resulted in a reduction of beneficial bacteria such as *Lactobacillus*, which was increased by l-THP treatment. These findings align with the mentioned reports.

The analysis of the relationship between serum metabolites and gut microbiota revealed an association between the disorder of the bacteria in Prevotellaceae_*NK3B31*_group and metabolite levels [H-Asn-Arg-Gln-Lys-OH; ((2-amino-3-((2-amino-3-((carboxymethyl)amino)-3-oxopropyl) dithio) propanoyl) amino) acetic acid; CMP-N-trimethyl-2-aminoethylphosphonate; docetaxel; D-name; pterocaryoside B; H-Val-Gly-Pro-Asp-OH; momordicoside L; prodigiosin; chenodeoxycholylglutamine; and lactarofulvene) in l-THP-treated rats. Reports demonstrated that Prevotellaceae*_NK3B31*_group exhibits properties of probiotics, including the ability to reduce inflammation and enhance the functionality of the intestinal barrier (Shang et al., 2021). Studies indicated that the administration of Kai-Xin-San led to a notable increase in the prevalence of Prevotellaceae*_NK3B31*_group, which has been linked to inflammatory responses, immune function, and neurodevelopment (Wang et al., 2024). Reports demonstrated that Prevotellaceae*_NK3B31*_group in the major depressive disorder group was lower than that in the control group (Yu et al., 2023). We found that the abundance of Prevotellaceae*_NK3B31_*group in the K + T group was higher than that in the KET group. Our results are consistent with previous reports.

The correlation analysis between urine metabolites and intestinal flora indicated a relationship between the disorder of metabolite harmaline and its associated microbial communities (*Candidatus_Arthromitus*, Prevotellaceae*_NK3B31*_group, unclassified_*Bacteria*, *Prevotella*, *Anaerotruncus*, and *UBA 1819*). Harmaline is a β-carboline alkaloid that was first isolated from the seeds of the *Peganum harmala* plant (Khan et al., 2013), and it has been linked to various central nervous system conditions, including addiction, anxiety, depression, and pain modulation. Research indicated that harmaline induces anxiety at lower dosages, whereas it exhibits anxiolytic properties when administered at higher doses (Wu et al., 2009). Furthermore, findings from preclinical studies ranging from planarians to rodents, which have demonstrated beneficial effects of harmaline when combined with various substances such as alcohol and amphetamine (Nunes et al., 2016), indicate a solid biological foundation for the therapeutic potential of these compounds in substance use disorders (SUDs).

Abuse of ketamine can lead to an increase in the release of DA in the brain, thereby causing a rewarding effect. L-THP acts as an antagonist of DA D1 and D2 receptors. Therefore, we speculate that l-THP may counteract KET addiction by regulating DA receptors and may also act through the gut microbiota and its metabolites to influence central DA signaling (via D1/D2 receptors) to counteract KET addiction. Our urine metabolomic results indicate that tyrosine metabolism is the main metabolic pathway after the administration of l-THP. Tyrosine undergoes hydroxylation to form L-3,4-dihydroxyphenylalanine (L-DOPA), which is subsequently decarboxylated to produce DA. When DA is present, dopamine β-hydroxylase catalyzes its conversion into norepinephrine (NE) and epinephrine (Juárez Olguín et al., 2016). We speculate l-THP affects the release of DA by influencing the metabolism of tyrosine.

The gut microbiota primarily communicate with the CNS through neurotransmitters such as GABA, DA, NE, 5-hydroxytryptamine (5-HT), and histamine (Strandwitz, 2018). DA synthesis in the gastrointestinal tract has been attributed to certain species within the genera *Bacillus* and *Serratia*. The administration of l-THP led to a decrease in *Bacillus* species, consequently reducing the release of DA. This result is consistent with the previous reports. Alterations in the expression of dopamine transporters and D1/D2 receptors in the striatum have been linked to changes in the gut microbial composition. Notably, specific members of bacterial genera including *Prevotella*, *Ruminococcus*, *Lactobacillus*, and *Bacteroides* have demonstrated the capacity to influence key components of the dopaminergic system—such as receptors, transporters, and downstream targets—exerting either enhancing or inhibitory effects (Cheon et al., 2021; Cao et al., 2019). We found that *Prevotella*, *Ruminococcus*, and *Lactobacillus* changed after the administration of l-THP. Therefore, we speculate that l-THP affects DA receptors by influencing the intestinal flora.

## 5 Conclusion

In summary, l-THP effectively relieved hippocampus pathological changes and attenuated KET-induced CPP in KET-abused rats. Serum and urine metabolomics identified 194 differential metabolites, including 3-methoxytyramine, 3-hydroxyphenylacetic acid, and phosphorylcholine, which were mainly involved in glycerophospholipid metabolism, tyrosine metabolism, and phenylalanine metabolism. L-THP also enriched *Firmicutes*, *Lactobacillus*, *Dubosiella*, unclassified_*Clostridia*_UCG_014, and *Ligilactobacillus.* Moreover, Spearman’s correlation analysis showed that discriminative metabolites were closely correlated with special bacteria. In this study, we preliminarily evaluated the impact of l-THP on both metabolomic profiles and gut microbiota in KET-abused rats and provided a basis for the prevention and intervention of l-THP on KET addiction.

## Data Availability

The data presented in the study are deposited in the NCBI repository, accession number PRJNA1358186.
